# Prevalence of Malaria

**Published:** 1878-06

**Authors:** 


### Miscellaneous.

### Prevalence of Malaria.

I would like to say, with regard to the prevalence of malarial diseases on different topographical areas, that it is a matter of observation, that such diseases prevail especially on the flat lands bordering on water-courses. I speak of the malarial regions of the South and Southwest, including Mississippi and Alabama; the same observation will apply to Southern Illinois, Missouri, Kansas and the States bordering on the Ohio, and also to some extent to Northern Illinois, likewise of the greater unhealthiness of the river and creek bottoms. The fact is so well known at the South that planters used to regulate their lives by it. They had often several plantations, one of which they took care to keep up, though less productive than the others, in the hilly districts. Between the Mississippi and Yazoo rivers the country is a dead level; the planters leave these lands for the hills, thirty or forty miles away, in summer, or rather used to do so before the war, where they could afford it. It is a prevalent custom at this time, for white laborers to leave the unhealthy bottoms in July. after having picked their crop of cotton, not to return to them again until September, at the earliest. It is from personal observation also that I affirm the level ground and swampy districts to be more unhealthy than the hills. This I believe to be so, not solely because the flats are generally marshy and more or less miasmatic of themselves, but because they are often situated at the base of eminences which bound them. We must recollect that from these eminences there is not only a pluvial drainage but an ærial one also; at night cooled air passes down to the levels, and carries with it the miasmatic effluvia of the hill sides. Every night the cold, humid air, charged with whatever miasm may be generated from the soil, rolls down upon the flats and remains there until the next morning, when the fog rises. Nothing is more firmly believed by the resident population, than what I have affirmed, indeed they would laugh any other opinion to scorn, and I am sure they know more about it than doctors who write on the subject in a scientific way, and never saw a case of congestive remittent in their lives, or had the least opportunity for studying the etiology of these semi-tropical maladies.

I must say, it has been a source of regret to me that during the last ten or twelve years the germ theory of disease has obtained so strong a food-hold in medical opinion. This theory has not been proved by any means; it is still a decidedly cloudy affair; a hypothesis which has never been established; a vague assumption imaginated by a class of thinkers who feel themselves unable to grope with the subject without having recourse to empirical hypotheses. Indeed, I am strongly inclined to believe that there is no such thing as the special agent we call malaria, and that the disease generally spoken of is due to so-called malaria are to a very much greater extent than has been supposed, the products of long continued and exaggerated heat and humidity combined. In this climate, we are not able to understand the intensity and exhausting effects of the heat and humidity of semi-tropical countries. There, all day long, for six or seven months of the year, from April to the last of October, or the first of November, the heat is excessive. The humidity is likewise exceedingly high; with regard to the temperature, higher during the early Autumn than in the middle of Summer. Now, *humidity* is *tantamount* to *heat*; every degree of humidity is equivalent in its effects upon the animal body to several degrees of heat, at least.

It is the humidity of the air that prevents the cooling of the body when over-heated. The constant excitement of the circulation, and the exaggeration of the nutrient processes generally, in consequence of the abnormally high temperature which the organism has to assume, eventually explodes in a febrile movement. But humidity and heat combined, the vastly greater portion of the so_

called effects of "malaria." Malaria is no single or peculiar emanation from the soil, capable by itself, of generating the disease in question. I do not by any means, however, wish to deny altogether the influence of animal and vegetable decomposition upon the origin of what we recognize as malarial affections. During the decomposition of both animal and vegetable matter gases are formed which are competent to deoxidize the blood. During the circulation of malarial diseases, also, I have no doubt that many other agencies of intra systemic origin, resulting from the overheating of the body, conspire to induce a spanæmic condition of the blood. If we draw blood in such diseases we kill our patients. This remark is especially applicable to the most malignant form of fever known at the South, viz: yellow fever, and is applicable, though less cogently, to all idiopathic fevers, for fever is indisputably an expression of hypo-oxygenation of the blood. Anything which induces poverty of the blood necessarily disposes to an attack of fever, so that I am satisfied that the influences of heat and humidity far more highly express than we usually understand in this latitude, are much more directly concerned in the generation of malarial fevers than has been generally taught.

The prevalence of malarial diseases in marshy places is due to the accumulated heat of the soil; the rays of the sun pass easily, by virtue of their high tension, through a humid atmosphere, and are absorbed by the soil, but the rays of low tension given off by the soil in return cannot traverse the overhanging humid air. This is a commonplace fact in meteoroogical thermics. Most transparent media act in the same way; thus in a hot house the rays received through the glass cannot pass out again, but are retained by the earth and herbage within, and so a high temperature is attained. A humid air, like the glass of a hot house, or the water overlying decaying leaves in a swampy pool, acts as a suntrap, and in all cases the earth becomes hotter than it would otherwise do. If the air is humid, the soil heat at night remains high, and houses and the human body must continue at an abnormal temperature, the system is then very soon overborne.—*Dr. Ford in St. Louis Medical and Surg. Journal.*

